# Systematic review for non-surgical interventions for the management of late radiation proctitis

**DOI:** 10.1038/sj.bjc.6600360

**Published:** 2002-07-02

**Authors:** A S Denton, H J N Andreyev, A Forbes, E J Maher

**Affiliations:** Center for Cancer Treatment, Mount Vernon Hospital, Rickmansworth Road, Northwood, Middlesex HA6 2RN, UK; Medicine & Therapeutics [Division of Medicine], Faculty of Medicine, Imperial College, Chelsea & Westminster Hospital, 369 Fulham Road, London SW10 9NH, UK; Chelsea & Westminster & Royal Marsden Hospitals, London, UK; St Mark's Hospital, Northwick Park, Watford Road, Harrow, Middlesex HA1 3UJ, UK

**Keywords:** radiotherapy, complications, proctitis, systematic review

## Abstract

Chronic radiation proctitis produces a range of clinical symptoms for which there is currently no recommended standard management. The aim of this review was to identify the various non-surgical treatment options for the management of late chronic radiation proctitis and evaluate the evidence for their efficacy. Synonyms for radiation therapy and for the spectrum of lower gastrointestinal radiation toxicity were combined in an extensive search strategy and applied to a range of databases. The included studies were those that involved interventions for the non-surgical management of late radiation proctitis. Sixty-three studies were identified that met the inclusion criteria, including six randomised controlled trials that described the effects of anti-inflammatory agents in combination, rectal steroids alone, rectal sucralfate, short chain fatty acid enemas and different types of thermal therapy. However, these studies could not be compared. If the management of late radiation proctitis is to become evidence based, then, in view of its episodic and variable nature, placebo controlled studies need to be conducted to clarify which therapeutic options should be recommended. From the current data, although certain interventions look promising and may be effective, one small or modest sized study, even if well-conducted, is insufficient to implement changes in practice. In order to increase recruitment to trials, a national register of cases with established late radiation toxicity would facilitate multi-centre trials with specific entry criteria, formal baseline and therapeutic assessments providing standardised outcome data.

*British Journal of Cancer* (2002) **87**, 134–143. doi:10.1038/sj.bjc.6600360 www.bjcancer.com

© 2002 Cancer Research UK

## 

Pelvic radiotherapy is an essential component of curative therapy for many cancers of the urological, gynaecological and gastrointestinal tracts. In the treatment of pelvic malignancy there is a need to minimise the risk of chronic radiation injury to normal tissue without compromising the possibility of cure. The gastrointestinal tract and in particular the rectum, is the site most frequently affected. The prevalence of severe toxicity such as fistulation, stricture formation, transfusion-dependent bleeding or secondary cancers after pelvic radiotherapy is unknown but has been estimated to be 5% at 10 years (Nostrant, 1995) although this may be an underestimate (Komaki *et al*, 1995; Tan *et al*, 1997; Denton *et al*, 2000). Much more common may be lesser side effects such as diarrhoea, urgency, faecal incontinence and tenesmus which in recent studies have been suggested to impair quality of life considerably (Kollmorgen *et al*, 1994; Schultheiss *et al*, 1997).

Acute radiation proctitis refers to radiation-induced injury of the rectum, during or within 3 months of radiotherapy. Chronic radiation proctitis can continue from the acute phase or begin after a latent period of at least 90 days (median time 8–12 months) (Eifel *et al*, 1995) and may be more common in those who develop severe acute proctitis (Denham *et al*, 1999), and in those with diabetes, inflammatory bowel disease, hypertension or peripheral vascular disease. Most of these risk factors have been defined by studies dependent on suboptimal assessment of rectal toxicity. Other factors which may increase the risk of late complications include tumour stage, and the total dose and fractionation of radiation, although the severity of radiation damage may not be entirely dependent on the radiation dose (Schwarchuk *et al*, 2000).

Whether a patient as developed chronic proctitis may be assessed in various ways. Changes in symptoms such as irregularity of bowel dysfunction, rectal blood loss or pain may be graded according to criteria stated in systems such as LENT SOMA and the Franco-Italian Glossary (Chassagne *et al*, 1993). The criteria for the grade of severity varies with the scoring system used, emphasising the need for a single universally agreed measure, but there are no described specific radiological features which define radiation proctitis (Capps *et al*, 1997). Endoscopically, proctoscopy, rigid sigmoidoscopy or flexible sigmoidoscopy may be used but it is recognised that these different methods may not produce reproducible findings between observers (Baron *et al*, 1964). Tissue biopsy may be inconclusive. The few small longitudinal studies of ano-rectal physiological parameters following RT that have been published are contradictory (Iwamoto *et al*, 1997; Yeoh *et al*, 2000).

In other inflammatory conditions affecting the bowel, symptoms and measures of disease activity may correlate poorly (Yoshida, 1999) and there has been little research to determine how endoscopic or radiological appearances relate to bowel function. Severe fibrosis with stricturing may provide incontrovertible evidence of radiation-induced damage but radiation-induced bowel changes are also seen in patients without symptoms and sometimes it can be hard to attribute the patients' bowel habit directly to physical damage caused by radiation to the rectum.

New gastrointestinal symptoms may be triggered for many reasons. Firstly, therefore, in the patient who has undergone radiotherapy, it may be difficult to untangle the relative contribution of psychological and physical damage to bowel function. Secondly, when there is severe physical radiation-induced damage present in the rectum, it is possible that patients will also have other sites of bowel damage, perhaps leading to bile salt or fat malabsorption. Thirdly, published series may not represent a true cross section of irradiated patients. There is some data suggesting that the reported number of cases with chronic radiation proctitis and a proportion of patients who seek help for subsequent symptoms is a fraction of the true prevalence (Yeoh and Horowitz, 1987; Ooi *et al*, 1999). Experience from both oncological and gastroenterology practice has long shown that unless directed questions are asked and exact answers pursued that patients may not tell their physicians about their specific gastrointestinal symptoms, particularly if they feel the physician is not in a position to do anything about them. The medical treatment of radiation proctitis is not clearly defined and management is often difficult. This is in part, due to problems establishing the diagnosis and also because a proportion of the biological changes may not be reversible.

The aim of this study was to examine systematically the non-surgical treatments that have been proposed for this condition and the quality of the evidence that suggests those treatments may be efficacious.

## MATERIALS AND METHODS

1. Criteria for considering studies for this review:
  *Types of studies*
    Randomised studies were included preferentially for analysis. Conclusions from the non-randomised, non-controlled data were drawn if there was insufficient evidence from randomised controlled trials.
  *Types of participants*
    Patients must have been diagnosed with a pelvic malignancy and undergone pelvic radiotherapy as part of their treatment schedule, subsequently developing radiation proctitis of any grade, continuing from completion of radiotherapy for more than three months, or occurring more than three months after completion of radiotherapy.
  *Interventions*
    Studies were included which described the use of non-surgical interventions for the treatment of late radiation proctitis and the specific intervention was used either *vs* a placebo/nothing or against other therapies.
2. Search strategy for identification of studies:
  *Concepts*
    - Synonyms for radiation therapy
    - Synonyms for the spectrum of lower gastrointestinal radiation toxicity
    - Concepts A and B combined with the Boolean operator ‘AND’
    A filter was not used because any type of study was considered. This basic strategy was expanded for text and MeSH terms before being applied to a sequence of databases. Relevant studies were identified and the inclusion criteria were then applied.
  *Databases*
    In order to be as comprehensive as possible search strategies were employed to identify all relevant studies irrespective of language. First, electronic databases were searched. The time frame used was from April 2001 back to 1966 (Table 1

**Table 1:**
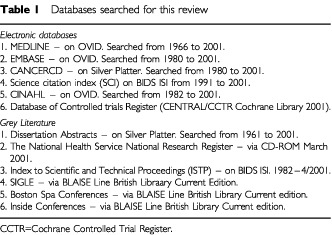
Databases searched for this review

). Secondly, reference lists of identified studies and the relevant chapters of major oncology and gastroenterology text books were searched. Thirdly, groups and individuals likely to hold unpublished data were approached (Table 2

**Table 2:**
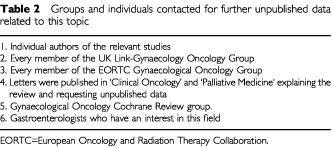
Groups and individuals contacted for further unpublished data related to this topic

).
3. Methods of the review:
  A final list of all potential relevant articles was created in one core database and were assessed independently by two reviewers to determine whether they complied with the preceding inclusion criteria for this the review. Where differences existed they were resolved by consensus and when necessary in consultation with a third reviewer. Included studies were graded according to the criteria used by the NHS executive (Table 3

**Table 3:**
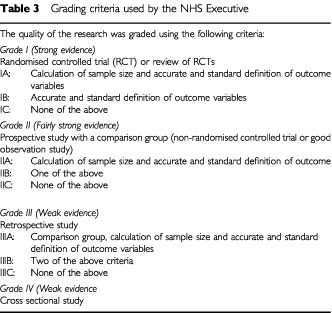
Grading criteria used by the NHS Executive

).
4. Statistics:
  Dichotomous data is expressed as the odds ratio. Uncertainty in each treatment is expressed using 95% confidence intervals (95% CI). Continuous data, i.e. symptom scores, were converted to the weighted mean differences and an overall weighted mean difference was calculated with standard errors. The Cochrane Review Manager software RevMan 4.1 was used for estimation of overall treatment effects/meta-analysis of results. Both fixed and random effects models were used to calculate a weighted average of the treatment effects across the studies under review. Sensitivity analyses as well as including study quality (quality of allocation concealment) also included year of publication, the type of outcome measures, and random and fixed effects models if appropriate.

## RESULTS

Using the search strategy described, studies were identified, of which 62 met the inclusion criteria, and included three randomised controlled trials (Table 4

**Table 4:**
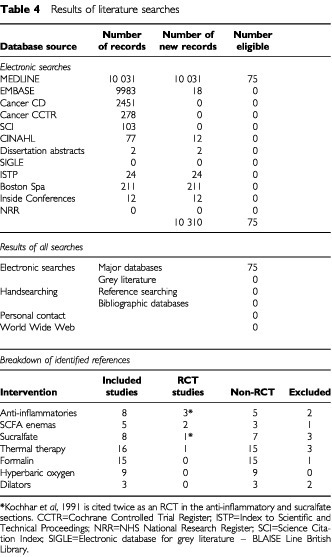
Results of literature searches

).

### Studies using anti-inflammatory agents

First line treatment of this condition is conventionally with anti-inflammatory agents. Seven studies were identified which used such agents. The highest level of evidence was provided by three randomised controlled trials (grade IC).

#### Kochhar *et al*, 1991, Grade IC, India

*Radiation-induced proctosigmoiditis. Prospective, randomised, double-blind controlled trial of oral sulfasalazine plus rectal steroids vs rectal sucralfate (*n*=37)* There were 36 females treated for cervical cancer and one male treated with prostate cancer. The mean duration from the completion of radiotherapy was 8.3 months. Exclusion criteria were the use of steroids in the last 2 weeks. Symptoms were assessed using an in-house scoring system for diarrhoea, bleeding, tenesmus and endoscopic appearance. According to the score the cases were then graded. Baseline characteristics in both groups were comparable. Patients were randomised to either, rectal prednisolone 20 mg b.d. and oral sulfasalzine 500 mg t.d.s. (*n*=18) or rectal sucralfate suspension 2 g b.d. with oral placebo t.d.s. for sulfasalzine (*n*=19). There were three drop-outs in the anti-inflammatory group and two in the sucralfate group. Treatment was continued for 4 weeks.

Eight out of 15 in the anti-inflammatory group showed clinical improvement compared to 16 out of 17 in the sucralfate group. Seven out of 15 in this group showed endoscopic improvement compared to 12 out of 17 in the sucralfate group, and the degree of improvement was greater in the sucralfate compared to the anti-inflammatory group. The odds ratio for clinical improvement was 14.0 (95% CI 1.46–134.26). No difference in endoscopic appearance was detected between the sucralfate and the anti-inflammatory groups, odds ratio 2.74 (95% CI 0.64–11.76). The response was only reported for the 4 week follow-up period. There was no quality of life assessment.

#### Rougier *et al*, 1992, Grade IC, France

*Rectites radiques: efficate comparee de deux types de corticoides adminstre localement (*n*=32) (Radiation proctitis: comparing the efficiency of local administration of two types of corticosteroids)* There were 29 females and three males, treated with radiotherapy for 23 gynaecological malignancies and nine colorectal or anal tumours. All had completed radiotherapy a minimum of 6 months ago. The diagnosis of radiation proctitis was graded on flexible sigmoidoscopy and on the degree of bleeding. Two rectal steroid preparations were compared, 5 mg betamethasone enema b.d. (*n*=16) or 90 mg hydrocortisone acetate mousse b.d. (*n*=16). The outcomes used were bowel activity, rectal bleeding, tenesmus with endoscopic grading. They were assessed clinically and endoscopically at 14 and 28 days. Two patients were lost to follow-up from the betamethasone group, and no treatment related complications were reported.

Over the 4 weeks of treatment the endoscopic appearance improved in a greater proportion of the hydrocortisone group (12 out of 16) than the betamethasone group (5 out of 14), odds ratio 5.40 (95% CI 1.12–26.05). There was no difference in the reduction of bleeding between the hydrocortisone group (6 out of 16) and the betamethasone group (3 out of 14), odds ratio 2.20 (95% CI 0.43–11.22). The response was only reported for the 4 week follow-up period. Potential bias in this study includes more severe intial disease in the betamethasone group and also that the betamethasone enema was poorly tolerated in 10 out of 14 compared with 2 out of 16 in the hydrocortisone group. There was no quality of life assessment.

#### Cavcic *et al*, 2000, Grade IC, Croatia

*Metronidazole in the treatment of chronic radiation proctitis: clinical trial (*n*=60)* Sixty patients with rectal bleeding and diarrhoea were allocated to treatment with metronidazole (3×400 mg orally per day), mesalazine (3×1 g orally per day), and betamethasone enema (daily) or mesalazine and betamethasone enema, but without metronidazole, for 1 year. The efficacy of metronidazole was assessed on the basis of rectal bleeding, diarrhoea, and rectosigmoidoscopy in all patients. The incidence of rectal bleeding and mucosal ulcers was significantly lower in the metronidazole group at 4 weeks (*P*=0.009), 3 months (*P*=0.031), and 12 months (*P*=0.029). There was also a significant decrease in diarrhea and oedema in the metronidazole group, after 4 weeks of treatment (*P*=0.044), 3 months (*P*=0.045), and 12 months (*P*=0.034). One year after treatment, 22 out of 24 of the metronidazole group demonstrated a reduction in the grade of their rectal bleeding compared to 5 out of 12 in the group treated with mesalazine and betmethasone, odds ratio 15.40 (95% CI 2.43–97.68). Similarly 23 out of 24 in the metronidazole group compared to 8 out of 12 had experienced reduction in their diarrhoea and rectal erythema, odds ratio 11.50 (95% CI 1.11–118.71). The rectal ulceration at 1 year had decreased in 22 out of 24 of the metronidazole group compared with 7 out of 12 of the group treated with anti-inflammatories alone, odds ratio 7.86 (95% CI 1.24–49.84). No side effects were reported.

#### Other studies

In addition, four non-randomised studies were identified. One prospective cross-over trial of six patients, 5ASA enemas *vs* betamethasone enemas, showed no significant benefit for either treatment (Triantifillidis *et al*, 1990). Three series reported the effect of various anti-inflammatory agents: sulfasalazine enemas nightly for 2–6 months in a group of four patients (Baum *et al*, 1989) were not effective. Oral sulfasalazine, for 1 year, in a group of four patients (Goldstein *et al*, 1976) demonstrated an improvement from the baseline symptoms as did daily oral oestrogen and norethisterone for 1 month in one case of refractory radiation proctitis (Wurzer *et al*, 1998).

### Studies using SCFA enemas

Short chain fatty acids (SCFA) are the main oxidative fuel of colonic mucosa and their use may be impaired in chronic radiation proctitis. SCFA are predominantly produced in the colon by anaerobic bacterial fermentation of non-absorbed carbohydrates, in dietary fibre. Butyric acid is the most important. SCFA also exert a trophic effect on the colonic mucosa by stimulating the physiological pattern of proliferation and promoting cellular differentiation. In the setting of radiation induced ischaemia, the associated mucosal atrophy may interfere with mitochondrial fatty acid oxidation so supplementation of SCFA in the form of enemas could overcome this deficiency and improve the energy supply to colonocytes. Moreover, the dilator effect of SCFA on arteriolar walls may improve mucosal blood flow. Five reports of four studies were identified in this section. Two were randomised studies.

#### Talley *et al*, 1997, grade IC (Australia)

*Short-chain fatty acids in the treatment of radiation proctitis: a prospective randomised, double-blind, placebo-controlled, cross-over pilot trial (*n*=15)* Two females and 13 males; 12 prostate, one cervix and two rectum, treated with pelvic radiotherapy a mean period of 12.2 months earlier were assessed using an in-house symptom score (six items: rectal pain, rectal bleeding episodes, quantity of blood, days of diarrhoea, number of stools and urgency). Endoscopic and histological scores were also calculated. The patients were randomised to receive either a normal saline placebo enema or a 60 ml enema containing 40 mM of butyrate administered twice a day. Each treatment was prescribed for 2 weeks with a 1 week washout period between treatments. Three patients failed to complete the course. The total symptom score at baseline ranged from 2–11 (median 5.5). There was a non-significant improvement in symptom scores on the active treatment, mean score 3.5 (range 3–5) compared with 4.5 mean score (range 3–6) for those receiving placebo. The results are published in the form of the median value (plus interquartile range) for the average placebo period and active period and the raw data is required from the authors before the results can be dichotomised. There was no quality of life assessment.

#### Pinto *et al*, 1999, grade IC, (Portugal)

*Short chain fatty acids are effective in short term treatment of chronic radiation proctitis. Randomised, prospective double blind, controlled trial (*n*=19)* Nineteen patients (one male and 18 females) with grade III chronic radiation proctitis were randomised to SCFA enema, 60 ml b.d. for 5 weeks (*n*=10) or a placebo (*n*=9). Baseline characteristics of each group were comparable. The specific outcome variables monitored for a period of 6 months were: adverse effects, number of days of rectal bleeding in the previous week and haemoglobin. The endoscopic appearance was scored for: hyperaemia and neovascularisation, friability, oedema and erosions by two independent assessors who were blinded to the treatment. Biopsies for mucosal DNA and protein content were also measured.

One patient from the SCFA arm and two from the placebo arm did not complete the trial. At 5 weeks the patients treated with SCFA had a significant reduction (4.4 to 1.4) in the number of days per week of rectal bleeding (*P*=0.001) whereas in the placebo group it fell from 5.1 to 3.4 (*P*=0.12). Haemoglobin levels at the end of the treatment period were higher in the SCFA group, 13.1±0.9 *vs* 10.7±2.1 g dl^−1^ (*P*=0.02) and the endoscopic scores had decreased significantly in both groups but were significantly lower in the SCFA group (*P*=0.02). Changes in DNA and protein concentration decreased in both groups but significantly more so in the placebo treated group (*P*=0.05). In long term follow-up two patients failed to complete the course from the placebo group because of severe bleeding leaving nine in the SCFA arm and five in the placebo group. At the end of the 6 months the endoscopic score was similar in the two groups. No adverse events related to either of the groups were noted and there was no quality of life assessment. As a consequence of the way the data has been reported we have presented this data as a weighted mean difference (WMD) which for endoscopic scores was 1 (−2.33 to 4.33), a statistically non-significant difference between SCFA and placebo. For the number of days of rectal bleeding per week at the end of the treatment period the WMD was −2 (−4.4 to −0.4), again a statistically non-significant difference, but perhaps showing a trend to less days of rectal bleeding with the SCFA enema.

#### Other studies

In addition, two non-randomised series were identified. Mamel *et al* (1995) (IIIC), in an open study of six cases using SCFA reported a significant improvement in clinical and mucosal response although this is not statistically assessed. Al-Sabbagh *et al* (1996) (IIC) in their prospective pilot study of seven cases demonstrated a significant improvement for rectal bleeding but not endoscopic appearances. The duration of the response is not stated other than for the treatment period of 4 months in Al-Sabbagh *et al* (1996). No toxicity was reported in any of the studies. Quality of life data were not presented.

Although there were two trials in this category using the same intervention against a placebo, they are not directly comparable due to differences in the outcome measurements and method of data presentation. Talley *et al* (1997) was a cross-over study and used a cumulative score encompassing the number of episodes of rectal bleeding per week. Pinto *et al* (1999) recorded the number of days on which rectal bleeding occurred. Although both sets of cases were scored endoscopically, this is not on the same scale so that the numbers are not comparable. Additionally, regardless of the different scales, because of the absence of individual patient data we were unable to dichotomise and combine the data. Talley *et al* (1997) reported no benefit from the use of SCFA (but the treatment period of 2 weeks was short), and Pinto *et al* (1999) also reported no significant improvement either endoscopically or on rectal bleeding. It is not surprising that neither of these trials yielded any significant results. These numbers are small for any type of study but especially for prospective, randomised ones and therefore statistically underpowered.

### Studies using sucralfate and pentosan polysulphate

Sucralfate is a highly sulphated polyanionic dissacharide. In this setting, its postulated mechanisms of action include stimulation of epithelial healing and the formation of a protective barrier. Pentosan polysulphate (PPS) is a synthetic derivative of a glycosaminoglycan which is present in the surface of the bladder, vessels and the gastrointestinal tract lining. The ability of PPS to reduce epithelial permeability and prevent adherence has been extrapolated to the large bowel. Eight relevant references were identified addressing the effect of these agents in chronic radiation proctitis. One is a RCT, two prospective open studies, and four studies describe the effect of rectal sucralfate in case reports or small series with one report of the effect of oral sucralfate.

#### Kochhar *et al*, 1991, Grade IC, India

*Radiation-induced proctosigmoiditis. Prospective, randomised, double-blind controlled trial of oral sulfasalazine plus rectal steroids* vs* rectal sucralfate (*n*=37)* The strongest evidence for the use of sucralfate comes from the evidence presented in this trial, which compares the use of anti-inflammatories with rectal sucralfate. Criticisms of this study are that the allocation concealment is not clear and there is no explanation for those cases lost to follow-up. The endpoints used to evaluate response, i.e. scores for clinical and mucosal effect, are useful and show that the odds ratio for the beneficial effect of sucralfate on clinical outcomes is 14.0 (95% CI 1.46–134.26) and 2.74 (95% CI 0.6–11.75) for mucosal response. The duration of follow-up was only 4 weeks.

#### Other studies

In addition seven non-randomised studies were identified. In two prospective studies Kochhar *et al* (1999), followed 26 cases treated with rectal sucralfate, and Grigsby *et al* (1990) described 13 cases treated with oral PPS, IIC. Both are well conducted with definitive outcomes of response and show a benefit for the use of rectal sucralfate for a period of 4 months and oral pentosan for 1 year, respectively. Of the retrospective reports (four describe the use of rectal sucralfate as a case report (Kochhar *et al*, 1988), and series of three, eight and three patients respectively (Ladas and Raptis, 1989; Kochhar *et al*, 1990; Stockdale and Biswas, 1997). All showed a benefit in both clinical and mucosal outcome, although the majority of these were not graded or scored to demonstrate significant differences. One report outlines the beneficial effect of oral sucralfate in established chronic radiation proctitis in three isolated cases for a minimum of 3 years (Sasai *et al*, 1998). Morbidity related to sucralfate was not reported and there was one case of a rash with oral pentosan. The treatment intervals were generally short and none provide any quality of life data.

### Formalin therapy

In radiation proctitis, vascular telangiectasia and non-healing mucosal ulceration, perhaps due to an underlying obliterative arteritis, may lead to severe recurrent haemorrhage. Formalin may sclerose and seal fragile neovasculature in radiation damaged tissues preventing further bleeding. Application directly to the mucosa produces local chemical cauterisation. It can stop bleeding by sealing the neovascularised telangiectatic spots and ulcers. The success of bleeding control is related to the accurate localisation and application of formalin to all the affected points.

We identified 15 references relating to the use of rectal formalin. Three were prospective case series (level IIC evidence) and 12 were retrospective reports (level IIIC). These reports are quite heterogenous (Table 5

**Table 5:**
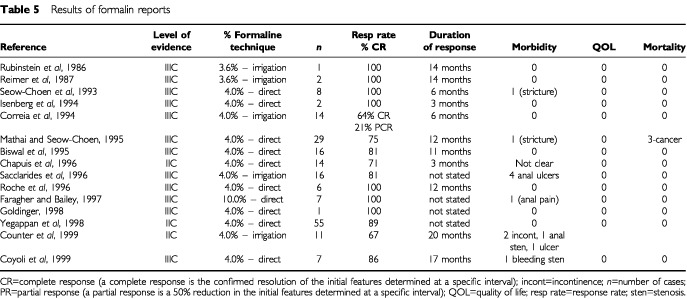
Results of formalin reports

). None used a control group or quoted any quality of life data. The severity of radiation proctitis is graded in only one report. The technique and the concentration of formalin used vary. The two main approaches use irrigation of formalin (five reports), either 3.6% formalin solution (*n*=2) or 4% formalin solution (*n*=3). The other method (described in 10 reports) is the direct application of gauze soaked in formalin, and although most reports used 4% formalin (*n*=9) there was one report that used 10% solution. None of the reports used an objective scoring system to assess the response to treatment, but two reports described the effect in terms of changes of haemoglobin level. The remainder described the response in terms of the transfusion requirements or the effect on bleeding. Statistical analysis was not presented in any of these reports.

In summary, 208 patients were described with a mean follow-up of 6 months. In each of the reports there appeared to be benefit from the use of formalin in chronic haemorrhagic proctitis, but this may be subject to reporting bias. Serious side effects were reported in 11 patients with five cases of anal ulceration, two rectal strictures, two patients with faecal incontinence and two with anal pain. Minor side effects were not frequently reported. Duration of effect cannot be assessed reliably from the data available but appears to be a minimum of 3 months. The absence of quality of life data means the impact of this treatment from the patient's perspective cannot be addressed.

### Thermal coagulation therapy

Endoscopic coagulation with a variety of devices has been reported to be effective for the control of radiation-induced bleeding. The technique generally used is coagulation of focal bleeding telangiectasia rather than the entire friable mucosa. Several treatment sessions are often required. Scarring and re-epithelisation with more normal tissue tend to occur over time.

Sixteen relevant studies were identified. Apart from one RCT and one prospective series the remaining 14 are retrospective series or reports. Although these case series all refer to the use of ablative therapy in late radiation proctitis, different types of coagulation probes or lasers are described. Eight look at the use of the YAG laser, four at Argon lasers, three at bipolar cautery and two at heater probes. The results of these studies are described in Table 6

**Table 6:**
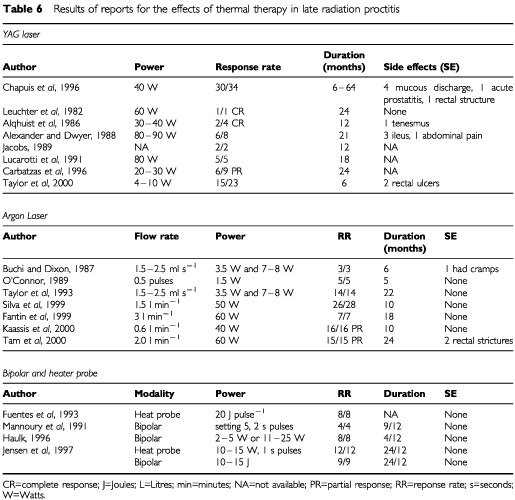
Results of reports for the effects of thermal therapy in late radiation proctitis

.

#### Jensen *et al*, 1997, (USA) grade IC

*A randomised prospective study of endoscopic bipolar electrocoagulation and heater probe treatment of chronic rectal bleeding from rectal telangiectasia (*n*=21)* Selection criteria included pelvic RT at least 2 years earlier, rectal bleeds at least three times per week, anaemia, having failed 1 year of medical therapy and consideration for surgery, and a life expectancy of 2 years. Chronic radiation proctitis was confirmed endoscopically but not graded in 21 cases (18 with carcinoma of the prostate and three with cervical cancer). Patients were randomised to treatment with a heater probe (*n*=9) or a bipolar electrocoagulation probe (*n*=12). Treatment sessions were repeated with the same probe till the bleeding resolved and a mean of four sessions per case were administered for both probes. The physicians who subsequently assessed the patients were blinded. All other treatments were discontinued. Assessment was repeateded every 4–6 weeks until bleeding stopped and then every 4–6 months for 1 year. If new telangiectasia were noted they were retreated by the same probe.

In both the bipolar probe and the heater probe the mean fall in severe bleeds per case was statistically significant at *P*<0.05. In the 12 months of endoscopic treatment *vs* 12 months medical therapy the severe bleeding episodes (defined as a bleed provoking and unscheduled hospital assessment) diminished significantly for the bipolar probe (75 *vs* 33%) and heater probe (67 *vs* 11%). The weighted mean difference did not show a significant difference between the two treatments 0.30 (95% CI −0.35 to 0.90). The reduction in the units of blood transfused per case pre- and post-treatment was only statistically significant in the group treated with the heater probe and the weighted mean difference reflected this (−3.2; 95% CI −4.58 to −1.82). The increase in the haematocrit compared with the baseline was statistically significant for both groups and comparing the effects of the two groups the weighted mean difference favoured the heater probe (−2.90; 95% CI −5.22 to −0.58). During follow-up endoscopy there was resolution of the telangiectasia, scarring or epithelial replacement in all cases in both groups. Patient interviews before, and 6 months after treatment revealed that rectal bleeds, tenesmus and their general health had improved. There were no recorded side effects.

#### Other studies

None of the references in this section state the grade of the proctitis and one study (Silva *et al*, 1999) uses a formal score to quantify rectal bleeding pre- and post-treatment allowing an objective comparison to be conducted. Generally, outcome assessments used are presence or absence of bleeding, control of bleeding, haemoglobin pre- and post-treatment and transfusion requirements. The point at which transfusion occurs is rarely described making transfusion requirements and haemoglobin values relative measures. The impression is that thermal coagulation therapy has a useful role in haemorrhagic radiation proctitis that is refractory to other medical treatments. This cannot be statistically supported because of the quality of the reports and the size of the trials.

### Hyperbaric oxygen therapy

Hyperbaric oxygen therapy (HBO) has an angiogenic effect and has been shown to cause an eight- or nine-fold increase in the vascular density of soft tissues over air-breathing controls. The subacute and chronic phases of radiation wounds are particularly suited to this form of therapy. HBO acts to stimulate collagen formation at the wound edges through elevation of local tissue oxygen tensions. New microvasculature dependent on collagen matrix, is greatly enhanced in this setting and allows re-epithelialisation to occur.

Nine relevant references were identified, reporting treatment in 86 cases that were followed up for a mean period of 15 months (Table 7

**Table 7:**
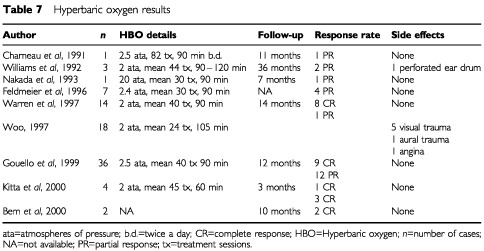
Hyperbaric oxygen results

). Eight were retrospective series or reports and there was only one prospective observational case series (Williams *et al*, 1992: level IIC). All of these reports were case series with heterogeneous characteristics. In only two of these reports (Feldmeier *et al*, 1996; Gouello *et al*, 1999) were there full baseline assessments of the degree of chronic radiation proctitis with a score or a grade of the histological or symptomatic features. Also the pressures of HBO and duration of the treatment sessions varied. The number of treatments depended on degree of the lesion. Assessment of response was usually with a vague description of the resolution of symptoms but not parameters that could be scored and used for statistical analysis. The duration of response was inconsistently recorded. Quality of life data was not recorded in any report. Side effects were reported in eight cases but were largely transient, minor and related to aural barotrauma. Therefore, although the impression is that HBO may be of value for large bowel chronic radiation changes that are refractory to other treatments, the degree of benefit and the cumulative effect or duration of response cannot be quantified because of the methodology and quality of the data.

### Miscellaneous interventions

Oxidative stress is thought to be a major mechanism in the development of chronic radiation proctitis and agents with anti-oxidant properties have been used in an attempt to limit tissue damage in radiation injury. We identified one series investigating the effect of vitamins C and E in the treatment of 10 cases with established chronic radiation proctitis who presented with one or more symptoms including rectal bleeding pain urgency or tenesmus. The severity and frequency of symptoms and a score of the lifestyle impact were used to assess response before and after treatment for 1 year and all reported a sustained improvement in their initial symptoms without side effects (Kennedy *et al*, 2001).

Strictures of the rectosigmoid junction and rectum are a recognised consequence of late radiation damage. The narrowing results in acute or episodic periods of large bowel obstruction, often decades after the original radiation therapy. Non-surgical dilatation has been attempted. Three relevant studies were identified and included two case reports of the use of a dilator (Triadafilopoulos and Sarkisian, 1990) or stent (Yates and Baron, 1999) and one series of four cases which were endoscopically dilated (Johansson, 1996). Although the strictures are described they are not scored and the absence of a formal baseline assessment and objective response means that the effect which appears to be beneficial in each report cannot be quantified, nor can the duration of response be determined from the data available. Side effects include one case of brief post dilatation bowel pain and another case of perforation. Quality of life issues are referred to in one report where the patients' general health was felt to improve as a result of the treatment (Yates and Baron, 1999).

## DISCUSSION

This review of the literature for non-surgical interventions for the management of late radiation proctitis has identified a number of treatment approaches supported by varying levels of evidence. Six controlled studies are reported, three relate to the comparison of different rectal steroid preparations and the comparison between anti-inflammatory agents and rectal sucralfate, two use short chain fatty acid enemas and one contrasts the effect of bipolar electrocoagulation *vs* the heater probe. These show that the use of sucralfate may be better than anti-inflammatory agents, which in turn may have greater effect if used with metronidazole. Rectal hydrocortisone may be better than betamethasone.

The reports describe slightly different interventions and outcome parameters so that they cannot be compared and a summary statistic cannot be derived. Ironically the wealth of case series although of interest, can only give an impression of effect and cannot be collectively summarised to produce a cumulative response rate or duration of effect. The overwhelming feature of the data presented here is that the majority of references describe one individual's or one centre's experience of a specific intervention administered without comparison to a control or another agent. In many cases, it is not clear how extensively patients have been investigated and whether other causes for their symptoms have been excluded. The reasons for the paucity of controlled studies may relate to the relative rarity of late radiation proctitis and the logistic difficulties that exist in compiling a series large enough to be randomised between therapies.

There were a number of specific problems with the details available in many of the identified reports. First, few background details were available with particular respect to the tumour stage and radiation details. Secondly, the method of determining the diagnosis was only occasionally documented. Late radiation proctitis may be less easy to grade accurately on rigid sigmoidoscopy compared to flexible sigmoidoscopy and there is also anecdotal evidence that some of the bowel preparations commonly used for flexible endoscopic examinations may exacerbate the changes seen. None of the endoscopic scoring systems take these factors into consideration but clearly the mode of assessment needs to be recorded for subsequent comparisons. Thirdly, there was rarely a formal baseline assessment of the symptoms and where this was stated the scoring systems were often different so that the grade could not be used as a comparison with other cases. Fourthly, the outcome measures, where used, were variable as was the scoring of the response when performed. The duration of the responses and the side effects of treatments were not always stated. Quality of life scores either before or after the intervention were hardly ever recorded. Deterioration in presenting symptoms may not always relate to failure of the intervention but may be a consequence of tumour or disease progression although this is often difficult to determine as the two scenarios are often indistinguishable. Therefore specific details regarding the method of ascertaining a failed response need to be stated. Finally, interventions were not standardised so that there were substantial variations in dose and administration. These combined factors serve to produce a very heterogeneous cohort of reports that cannot be scored or graded to produce subclasses and objective response rates unless more information is available. A proposed scheme to address all these issues might include:

1. A detailed account of the determination of the diagnosis including background details such as precise radiation prescription and absence of infection.
2. Scored symptoms as in Ulcerative Colitis (UC) with the Colitis Activity Index (Rachmilewitz, 1989) or the Ulcerative Colitis Scoring System (Schroeder *et al*, 1987), before and after the specific intervention.
3. Scored endoscopic appearance (as for UC e.g. Baron score (Baron *et al*, 1964) and the Colitis Endoscopic index (Rachmilewitz, 1989)), before and after the specific intervention.
4. Scored histological assessment (as for UC) which should be standardised.

The outstanding issue is the degree to which radiation induced damage to the rectum contributes to symptoms in this group of patients and how much comes from residual tumour, psychological factors, co-incident small intestinal damage or other changes within the abdomen and pelvis outside the bowel. Some of the areas that need to be addressed include:

1. How should late radiation proctitis be staged and how should endoscopic, radiological, physiological and quality of life assessments be combined to assess the patient's needs best?
2. Who needs intervention?
3. What treatments are effective?
4. Is there an optimal step up treatment approach?
5. How should patients be followed up after treatments, how long for and what parameters should be measured subsequently?

### Implications for practice and research

If the management of late radiation practice is to become evidence based then good quality placebo-controlled studies need to be conducted to support the treatment options recommended. This review suggests that late radiation proctitis is not reported very often by patients to the clinicians who deliver the pelvic radiotherapy and as a result a number of fundamental issues remain to be clarified. First, the true incidence of the disease is not clear. Therefore, physicians caring for patients who have undergone pelvic radiotherapy need to be more aware that this group may develop problematic symptoms which may need detailed questioning to elicit and which may require specialist gastroenterological assessment to characterize in detail. Secondly, there is an urgent need to define clearly the diagnostic criteria and a unified grading system by which late radiation proctitis can be categorised. Without such a system, it is unlikely that meaningful randomised studies, particularly in a multi-centre setting, can be designed.

We propose that cases should be enrolled into regional or centralised registers of radiation toxicity or that all such patients should be referred to regional centres with an interest in radiation-induced gut toxicity. In this way terminology, baseline assessments including comorbidity and the documentation of therapeutic effect could be standardised. Interventions could be randomised and outcome data could be pooled to assess the response to treatment objectively. This approach would provide an evidence base of results of different treatments to develop an integrated care pathway for this difficult condition.
